# Pre-clustering data sets using *cluster*4*x* improves the signal-to-noise ratio of high-throughput crystallography drug-screening analysis

**DOI:** 10.1107/S2059798320012619

**Published:** 2020-10-16

**Authors:** Helen M. Ginn

**Affiliations:** a Diamond Light Source Ltd, Didcot OX11 0DE, United Kingdom

**Keywords:** clustering, fragment screening, heterogeneity, software

## Abstract

Drug and fragment screening at X-ray crystallography beamlines has been a huge success. However, it is inevitable that more high-profile biological drug targets will be identified for which high-quality, highly homogenous crystal systems cannot be found. With increasing heterogeneity in crystal systems, the application of current multi-data-set methods becomes ever less sensitive to bound ligands. In order to ease the bottleneck of finding a well behaved crystal system, pre-clustering of data sets can be carried out using *cluster*4*x* after data collection to separate data sets into smaller partitions in order to restore the sensitivity of multi-data-set methods. Here, the software *cluster*4*x* is introduced for this purpose and validated against published data sets using *PanDDA*, showing an improved total signal from existing ligands and identifying new hits in both highly heterogenous and less heterogenous multi-data sets. *cluster*4*x* provides the researcher with an interactive graphical user interface with which to explore multi-data set experiments.

## Abstract 2

Separating multi-data sets based on C^α^ differences can help to identify additional hits for drug- and fragment-screening crystallography experiments, expanding the range of crystal systems to which this technique can be applied. This is made available in a graphical user interface.

## 1. Introduction   

Potential ligands are either soaked into pre-formed crystals or co-crystallized with their targets for X-ray diffraction data collection in drug- and fragment-screening experiments, which have been developed on several beamlines, such as XChem, developed by Diamond Light Source in collaboration with the Structural Genomics Consortium (Whitman, 2018 ▸), and the pipeline at the BESSY MX beamlines (Schiebel *et al.*, 2016 ▸; Wollenhaupt *et al.*, 2020 ▸). Recent advances in detectors, robotics and beam optics (Grimes *et al.*, 2018 ▸) have helped to fully realize the potential of the concept of fragment screening (Blundell *et al.*, 2002 ▸), and more beamlines are expected to specialize in high-throughput screening over the next few years (Förster & Schulze-Briese, 2019 ▸).

Modern screens produce a number of related individual data sets, known as multi-data sets, each of which must undergo data reduction and model refinement. These multi-data sets commonly have hundreds or thousands of individual members. Multi-data-set methods extract information from the plurality of data sets to inform analysis of the individual data sets. For example, one such method performs a statistical characterization to enable comparison across all collected data sets, thereby allowing the identification of a signal over background noise in electron-density maps (a hit). This method is implemented in the software package *PanDDA* (Pearce *et al.*, 2016 ▸). This software overcomes significant drawbacks in 2*mF*_o_ − *F*_c_ and *F*_o_ − *F*_c_ maps, where phase and overfitting biases can completely wash out any electron density associated with a hit. In these situations the ligand can often be clearly identified by *PanDDA*. *PanDDA* calculates the mean and standard deviation on a per-voxel basis across a multi-data set (the statistical characterization step) and produces event maps where voxel values are expressed in terms of standard deviations from the mean (the *Z*-map and event-map calculation step). Peaks which register above a certain *Z*-value are expanded by connecting them to neighbouring voxels above a minimum *Z*-value. Those which pass a minimum size threshold become potential hits for manual inspection. *PanDDA* has been effective in enabling ligand identification in a range of crystallographic screens (Keedy *et al.*, 2018 ▸; Glöckner *et al.*, 2020 ▸; Douangamath *et al.*, 2020 ▸).

Although *PanDDA* includes some realignment of maps according to C^α^-position variations, broad structural differences caused by crystal heterogeneity will diminish the signal-to-noise ratio by widening the distributions of individual voxels. To sidestep this problem, the focus is currently on obtaining a good crystal system in the first place rather than exploiting downstream processing methods, which has been described as the bottleneck (Collins *et al.*, 2018 ▸). This paper shows that providing *PanDDA* with pre-clustered data sets, where these variations are minimized within the sets, can enhance the power of the *PanDDA* method.

Choosing the members of each cluster is a similar problem to ensuring that data from multiple crystals are only merged if they are relatively isomorphous, which has also been tackled using hierarchical clustering (Giordano *et al.*, 2012 ▸). Another hierarchical method for grouping the most similar data sets has been developed in the computer program *BLEND* (Foadi *et al.*, 2013 ▸).

The most related method to that used in *cluster*4*x* is the *XSCALE_ISOCLUSTER* module in *XDS* (Diederichs, 2017 ▸). This is based on the correlation between absolute intensities in reciprocal space, and therefore gives an indication of the relative closeness of data sets, as well as the identification of clusters, based on a previous algorithm for ensuring uniformity of indexing choice for X-ray free-electron data snapshot images for space groups with an indexing ambiguity (Brehm & Diederichs, 2014 ▸). The Brehm and Diederichs algorithm introduced the concept of using an *N*-dimensional vector to represent each data set. The angle between two of these vectors, after clustering, has an inherent meaning: two data sets with a correlation coefficient of zero between them would have vectors at right angles with respect to the origin, and two data sets with a correlation coefficient of one would have a corresponding angle of zero degrees. However, variations which are small enough to fall within the level of the noise, but which may still have an impact on multi-data-set analyses, may go unnoticed, making it difficult to distinguish clusters by eye. The underlying methods for the clustering analysis presented in *cluster*4*x* rely on correlation between differences in reflection amplitudes or model C^α^ positions, rather than their absolute values, and therefore the ability to identify subtle clusters by eye is enhanced, at the expense of highlighting the magnitude of the differences between them.

Another modification of the underlying original algorithm for breaking indexing ambiguities (Brehm & Diederichs, 2014 ▸) is implemented in *dials.cosym* (Gildea & Winter, 2018 ▸), not only to break the ambiguity, but also to identifiy the indexing ambiguity itself by the inclusion of all potential symmetry operations leading to merohedral twinning in a given lattice type. The lack of prior assumptions about the lattice symmetry makes this particularly suited to automatic processing pipelines.

For the *cluster*4*x* clustering methods reported in this paper, although the detection and breaking of indexing ambiguities is possible, the focus is on identifying subtle variations that are found within a consistent indexing choice and do not necessarily have boundaries that are as clear-cut. The choice of clustering is manual and is powered through a graphical user interface (GUI), but is not a time-consuming or labour-intensive process, and provides plenty of opportunity for researchers to become acquainted with the peculiarities of their sets of crystals. Clustering using this method does not have to be limited to drug or fragment screens, but could be applied to the partitioning or verification of induced crystal changes for a wide range of additional variables.

## 2. Materials and methods   

### 2.1. Data acquisition   

The data sets for PTP1B (Keedy *et al.*, 2017 ▸) from a fragment screen (Keedy *et al.*, 2018 ▸) and for BAZ2BA, BRD1A and JMJD2DA (Krojer *et al.*, 2017*a* ▸,*b* ▸,*c* ▸) deposited with the original paper reporting *PanDDA* analysis (Pearce *et al.*, 2016 ▸) were downloaded from Zenodo (https://zenodo.org).

### 2.2. Generating average sets   

Average data sets were generated from either reciprocal-space reflection amplitudes or real-space C^α^-atom positions. A default but alterable resolution cutoff of 3.5 Å removes reflections beyond this limit from the analysis. This default was chosen to balance the speed and quality of clustering results. If multiple conformations of one C^α^ atom are present, only the first C^α^ conformer is used. Each multi-data set has *N* data sets. Each data set *n* has *I* reflections with amplitudes *F*_*i*,*n*_. For every reflection *i*, *N_n_* amplitudes have been recorded and *N* − *N_n_* amplitudes are missing from the data set. An average data set is generated, comprising *N* reflections, each of which with an amplitude 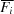, where 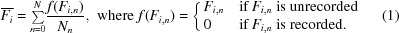



Each data set has an associated model with *J* C^α^ atoms with 3D coordinate vectors **c**_*j*,*n*_ in real space. *J_n_* atoms in data set *n* have been modelled for every C^α^ atom *j*, and *J* − *J_n_* atoms remain unmodelled. Similarly, an average model is generated with *J* C^α^ atoms, each of which with a coordinate vector 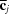, where
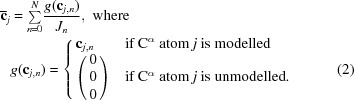



As one may not want to guarantee that all of the entered data sets will be of the same space group, this is not restricted to any asymmetric unit.

### 2.3. Scaling data sets   

A scaling step is carried out on each individual data set to remove the effect of any global isotropic *B* factors on downstream comparisons. Reciprocal space is divided into 20 equal volume bins, with concentrically spherical boundaries centred on the origin, and the diameter of the final bin equal to the d* value of the furthest recorded reflection amplitude (in Å^−1^). Each bin has a list of *B* reflection indices, *b*_1_, *b*_2_, …, *b* *_B_*, which point to a subset of all *I* reflections. For every data set, each bin only has *B* *_n_* recorded reflections, and *B* − *B* *_n_* unrecorded reflections. For each data set *n* and for every bin (not enumerated), a scale factor *k* is derived. Each amplitude *F*_*i*,*n*_ in this bin is then multiplied by *k*,
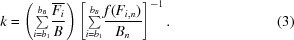



### 2.4. Pairwise correlation coefficients   

Correlation coefficients are calculated between series of values associated with data sets *m* and *n*, which are used in downstream analysis. For comparison in reciprocal space, spanning only amplitudes *F*_*i*,*m*_ and *F*_*i*,*n*_ recorded in both data sets, the series of values are
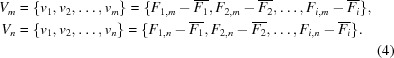



For comparison of C^α^ positions, spanning only vectors **c**_*j*,*n*_ and **c**_*j*,*m*_ modelled in both atomic models,
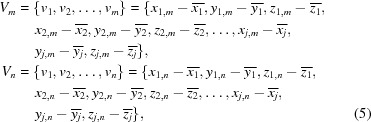
where
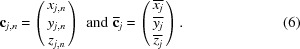



A Pearson correlation coefficient *a*_*m*,*n*_ was calculated between values series *v_m_* and *v_n_*, and bounded to a value between 0 and 1.

### 2.5. Clustering analysis   

A matrix **M** was prepared with *N* × *N* rows and columns. Each element **M** ^*n*^_*m*_ where *m* ≠ *n* was set equal to *a* ^*n*^_*m*_; where *m* = *n*, **M** ^*n*^_*m*_ was set to zero. Singular value decomposition (SVD) was then performed on this matrix,
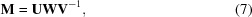
where **U** and **V** are orthogonal, and **W** is a diagonal matrix with positive or zero elements.

In the GUI, the researcher is presented with the *N* values of the **W** diagonal entries. The researcher is allowed to choose the three axes to display from a choice of **W** axis values; those with larger values encompass more of the variation seen in the data. If entries *n*_1_, *n*_2_ and *n*_3_ are picked, a submatrix **S** formed of *N* × 3 rows and columns is formed,
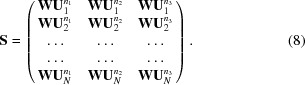



A three-dimensional plot is populated with *N* vectors, each of which has elements equal to each row of **S**. Each of these vectors represents the association of a single data set with the three selected clusters *n*_1_, *n*_2_ and *n*_3_.

### 2.6. Subclustering   

Structures which deviated significantly from the C^α^ positions could easily be identified and were removed from the clustering analysis; this was only required for the multi-data sets PTP1B and BRD1A. For each PTP1B structure, the appropriate symmetry operation was applied to bring all C^α^ positions to a common average position. On the removal of outliers and the application of symmetry operators, the C^α^-position averages could be recalculated without bias from outliers. Subclusters were selected manually using both the real-space and reciprocal-space clustering results as a guide. This was performed by rotating the three-dimensional SVD plot and either adding or subtracting from a selection using keyboard modifiers and clicking and dragging with the mouse. This required a few minutes to complete the clustering per data set. Sometimes, clusters were separated from the main group and clustering was rerun on these using either re­calculated sets of averaged structure factors and C^α^ atoms or using the original averaged sets. This allowed the finer separation of subclusters, should some data sets further from the mean exhibit significant further internal variation, by recalculating a new average. Alternatively, clusters could be marked as complete if they were deemed to require no further subdivision.

### 2.7. *PanDDA* analysis   

The output from clustering was organized into separate runs and the *pandda.analyse* module from *PanDDA* version 0.2.14 (Pearce *et al.*, 2016 ▸) included with *CCP*4 version 7.1 (Winn *et al.*, 2011 ▸) was executed on these partitioned data sets and also on the unpartitioned data sets. In both cases, this was run with the nonstandard parameter min_build_datasets=20, but otherwise with the default parameters. Event maps were inspected manually using *pandda.inspect*, with unclear results not reported in the original studies being re-evaluated, and new event maps evaluated by eye to determine whether they were true hits or whether the electron density was not clear enough. The criteria that a hit was considered to be a bound ligand were as follows: after the exclusion of backbone rearrangements, side-chain flips, water-molecule rearrangements and ions, the event-map density at the background-corrected sigma level of 1.0 had to cover the entirety of the ligand when modelled into the density or, for low-resolution structures, cover the vast majority of the ligand and leave little room for interpretation as one of the other excluded events. For BAZ2BA, JMJD2DA and BRD1A, hits were ignored if they were clearly present in both data sets, even if they were not reported in the initial study, including some ligands that were not modelled in the original analysis as they lay between nonphysiological contact sites. Owing to the fact that all original hits could be prescribed to two clusters in PTP1B, the 18 PTP1B clusters without any hits from the original analysis were not subject to this restriction.

## 3. Results   

For a total of *N* data sets, pairwise correlations between difference data sets were calculated, and so every data set was described using a vector of *N* scalar coefficients. Singular value decomposition (SVD) is a linear algebra technique which can draw out the accessible subspace of a matrix. This subspace is the possible range of vectors which can be reached through well defined linear combinations of the component axes of a matrix. SVD produces a set of orthogonal axes, weighted by their relative contribution to the accessible subspace. If there is some concerted behaviour of several data sets that behave in similar ways with respect to the average data set (*i.e.* having more similar correlation vectors), this is indicative that these should be combined into a cluster. SVD will therefore output a single heavily weighted subspace axis which describes this concerted variability. Axes associated with smaller weights represent more minor variations between data sets, and sufficiently small weights can be ignored. Although there are *N* orthogonal axes output from SVD, only a handful of these will have a large weight associated with them. The ratio between weights is important, rather than their absolute values. This clustering method can be carried out using either the deviation in the reflection amplitudes or the deviation in C^α^ positions from refined structures, or, owing to the interactive nature of the GUI developed to aid the application of this algorithm, a mixture of both.

A large multi-data set from a fragment screen of PTP1B (Keedy *et al.*, 2017 ▸, 2018 ▸) and three smaller publicly available multi-data sets published with the original *PanDDA* study (BAZ2BA, JMJD2DA and BRD1A; Krojer *et al.*, 2017*a* ▸,*b* ▸,*c* ▸) were downloaded. Additional processing results for the PTP1B study were kindly provided by Daniel Keedy, and for the three smaller multi-data sets *pannda.analyse* was used to recalculate the event maps and *Z*-maps (here referred to as the unpartitioned analysis). Alternatively, multi-data sets were divided into clusters using the *cluster*4*x* GUI before executing individual *pandda.analyse* runs on the clusters (pre-clustered analysis). The default parameter min_build_datasets, which usually requires 40 data sets at a minimum resolution to be reached for further processing, was lowered to 20 data sets in order to compensate for the reduced number of data sets in each cluster. An increase in noise in the statistical characterization may be offset by increased homogeneity in the selected clusters. The least homogenous multi-data set is PTP1B, for which *cluster*4*x* facilitated a dramatic improvement in the ligand-identification rate. The three smaller data sets contain fairly homogenous crystals; however, *cluster*4*x* is still capable of identifying additional hits in the screens. These smaller multi-data-set fragment screens are considerably smaller than what is routinely achieved following improvements in high-throughput methodology.

**Figure 1:**
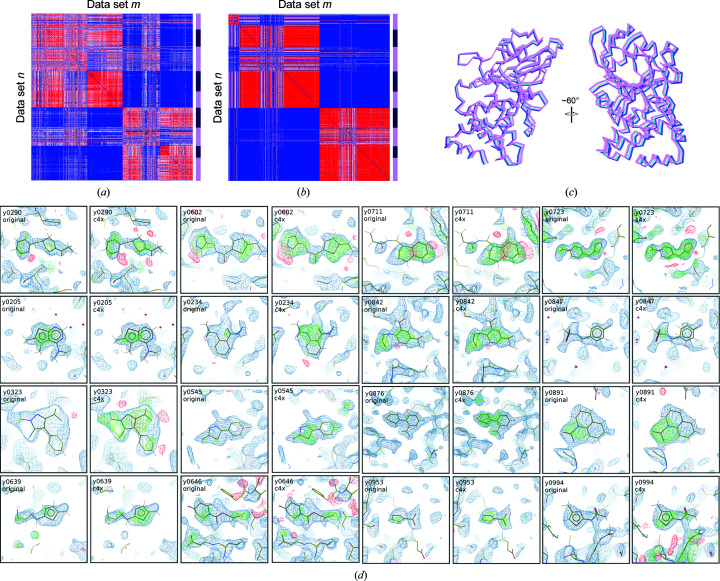
Cluster outcomes for multi-data set PTP1B. (*a*) Correlation plot showing relationships between data sets in reciprocal space according to Section 2 for clusters 1–9 in that order. The colour scale ranges from blue (coefficient of 0) through white (coefficient of 0.5) to red (correlation of 1). Alternating dark/light boundaries down the right-hand-side bar are a guide to the cluster boundaries. (*b*) Similar correlation plot based on C^α^ differences. (*c*) Two views of C^α^ positions for structures from clusters 2 (light blue), 3 (purple) and 6 (pink). (*d*) Views of 16 of the newly identified ligands, chosen from clusters 1–9 where the data-set number is less than y1000. *PanDDA* background-corrected event maps are displayed from the pre-clustered analysis in all cases (2σ), as these were often not calculated for maps dropping below the *Z* threshold in the unpartitioned analysis. *Z*-maps were available for both analyses in all cases, and so the corresponding map is displayed with positive values in green and negative values in red (±3σ). Electron-density figures were rendered in *Coot* (Emsley *et al.*, 2010 ▸).

PTP1B was the most populous multi-data set, with 1626 paired reflection lists and atomic models, and exhibited the highest variability. Data sets were first clustered on reciprocal-lattice amplitudes (resolving an inconsistency in the indexing ambiguity choice) into two major groups and were then further subclustered into 20 data sets using C^α^ differences, after collapsing the coordinates of all structures onto each other via applying the appropriate symmetry operator. For one of the resolved indexing choices, the correlation matrix was re-ordered by cluster and redrawn with a recalculated average. The correlations for amplitude differences (Fig. 1 ▸ *a*) and C^α^ differences (Fig. 1 ▸ *b*) show a divide into two major subclusters (clusters 1–5 and clusters 6–9), after which more subtle variations were extracted. For the other indexing choice there were more data sets, and therefore slightly more sub­divisions could be supported (nine versus 11). The C^α^ positions for clusters 2, 3 and 6, which were chosen for their distinct translational and rotational shifts, are shown in Fig. 1 ▸(*c*), showing the significant variability that can arise.

The resolution, unit-cell dimensions, *R*_work_/*R*_free_ and hit information for each cluster are shown in Table 1 ▸. The original study (Keedy *et al.*, 2018 ▸) identified 380 putative hits, of which 110 were accepted. The 110 original hits were concentrated into only two subclusters (one from each indexing choice) comprising 117 structures from clusters 1 and 10. These had significantly lower *R*_work_ and *R*_free_ values (18.8% and 21.7% on a background of 25.5% and 27.9%, respectively) and were distinguishable in the C^α^ positions in real space owing to an allosterically active alternative conformation in the N- and C-termini. They also had the highest average resolution (below 1.8 Å). There were no original hits in any of the other 18 clusters. The original study was executed on all data sets together, but *PanDDA* still groups structures by resolution range to avoid Fourier truncation errors. The likely explanation for the skewed pattern of hits is that this grouping by resolution acted as a pseudo-clustering which would have enriched the number of structures from clusters 1 and 10 analysed together in the highest resolution bins. A secondary effect from the significantly lower *R*_work_/*R*_free_ values would also increase the clarity of the event maps and the signal to noise of the *Z*-maps. When the original analysis examined lower resolution structures, structures from a wider range of clusters would be combined and the signal-to-noise ratio would reduce.

Pre-clustered analysis with *PanDDA* resulted in 472 hits in total. There were only two additional hits within clusters 1 and 10 together. However, across the clusters in which identified ligands were absent in the original analysis, an additional 74 hits were identified, together increasing the number of identified hits by 69% across the whole multi-data set. Changes in the signal level in the calculated *Z*-maps for many of the identified ligands within clusters 1–9 are shown in Fig. 1 ▸(*d*). *PanDDA* reports two values for clusters of voxels (here termed peak-clusters) characterized as hits: the mean *Z*-value of the peak-cluster and the peak-cluster volume in Å^3^, which is the total volume of the peak-cluster extending above the minimum peak value of *Z* = 2.5. One can calculate an estimate of the total signal for ligands shared between both runs by multiplying the peak-cluster volume by the mean *Z*-value. For data sets where a single putative hit was shared between the unpartitioned and pre-clustered analyses, the total signal increased by 15%, and was broken down into an increase of 18.4% in the the peak-cluster volume and a reduction in the mean *Z*-value of 3.5%, although the pre-clustered mean *Z*-value is calculated over a larger number of voxels and is therefore not strictly comparable. This suggests that pre-clustering produces broader peaks rather than higher peaks in the *Z*-maps. Note that this comparison does exclude a subset of weaker hits only identified in the pre-clustered analysis.

The *PanDDA* analysis of the unpartitioned data sets for the three smaller multi-data sets reproduced similar results as in the original study (Pearce *et al.*, 2016 ▸) as viewed using *pandda.inspect*. Small differences will be attributable to the change in the min_build_datasets parameter from the default. The list of putative hits is a mixture of events such as clearly bound ligands, unclearly bound ligands, backbone rearrangements, catalytic events, side-chain flips, bound ions, solvent fluctuations and false hits owing to statistical error rather than true density variation. Events in all but the first category are discarded. As for the PTP1B multi-data set, discarded events significantly outnumber those which are accepted as identified hits. False positives resulting from statistical error cannot be easily distinguished from true positive results where poor binding has led to unclear electron density. The same inspection was carried out on each of the pre-clustered analysis outputs. If a potential plausible ligand was identified but was present in both the pre-clustered and unpartitioned analyses, it was not included in the list of additional hits from *cluster*4*x*.

**Figure 2:**
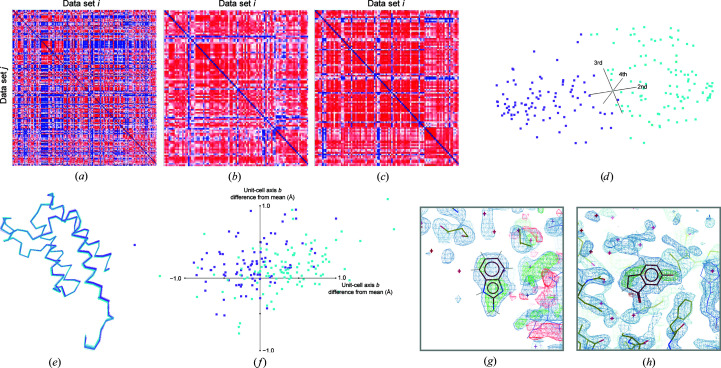
Cluster outcomes for multi-data set BAZ2BA. (*a*) Matrix plot showing relationships between data sets in reciprocal space according to Section 2. The cluster was separated in reciprocal space into two groups. (*b*, *c*) Matrix plots for subgroups plotted against the same average values calculated from all data sets. (*b*) corresponds to cluster A and (*c*) corresponds to cluster B in the main text. (*d*) Plot showing the second, third and fourth major axes of the SVD plot, showing separation of the two groups, which could be manually subdivided by splitting across the second axis. (*e*) shows separation in real space as a result of these reciprocal differences, plotting all C^α^ atoms in the structure. Blue corresponds to the data sets in cluster A and purple denotes those in cluster B. (*f*) Unit-cell deviations in the *a* and *b* axes from the average values across all data sets. (*g*, *h*) *PanDDA* background-corrected event maps in blue (2σ) and *Z*-map with positive values in green and negative values in red (±3σ) for (*g*) a newly identified hit from x447 and (*h*) a newly identified hit from x557.

The BAZ2BA multi-data set comprised 199 data sets for a small four-helix bundle. The protruding N-terminus lay alongside the equivalent from one of the symmetry mates, and the longer loop region between the first and second helices sat against the corresponding loop of another symmetry mate. This was pre-clustered using *cluster*4*x* before downstream analysis with *PanDDA*. Owing to the small number of data sets, this was divided into only two major clusters: A (101 data sets) and B (98 data sets) (Figs. 2 ▸ *a*–2 ▸ *c*). Clustering was easily carried out in reciprocal space with no need to separate on C^α^ positions, as this produced similar results. Fig. 2 ▸(*a*) shows that for a significant number of data sets, the deviations from the average of all members of the multi-data set show no net positive correlation with other data sets, which is coloured in blue on the diagram. Data sets which do not correlate well with one another are separated into separate clusters, which is why Figs. 2 ▸(*b*) and 2 ▸(*c*) have a reduced proportion of blue (zero) entries in the diagram. The properties of the two clusters are shown in Table 2 ▸.

**Figure 3:**
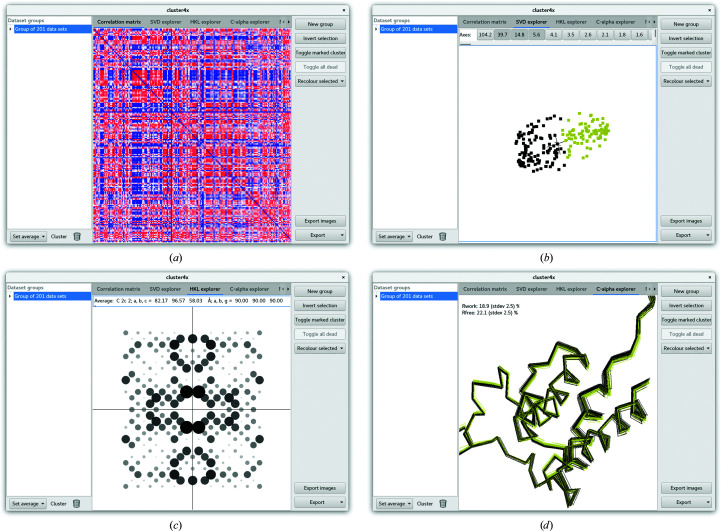
Screenshot of the *cluster*4*x* GUI. (*a*) View of the correlation-coefficient matrix plot after clustering. Clusters are listed down the left-hand column, which will also be populated with subclusters. Controls for generating new clusters are displayed on the right. (*b*) Rotatable SVD plot, with clusters selected manually by clicking and dragging to either add (+ Shift key) or subtract (+ Ctrl key) rendered in yellow. (*c*) Rotatable *hkl* space for viewing the amplitudes and unit cell of a cluster, which can also be rendered per data set, which is good for identifying mis-indexing results. (*d*) View of all C^α^ atoms, including those selected in (*b*) rendered in yellow.

Data sets were separated manually in *cluster*4*x* according to the SVD output (Fig. 2 ▸ *d*). In real space, the two clusters showed a shifting of the four-helix bundle as a rigid unit, while part of the N-terminus (residues 1857–1859) and the longer loop (residues 1893–1908) forming the crystal contacts remained anchored against their neighbours (Fig. 2 ▸ *e*). As changes in the internal motions of the protein will be accompanied by adjustment of the unit-cell dimensions to compensate, this will then also be correlated with adjustments in the reciprocal-lattice amplitudes (with the unusual exception of the protein expanding and contracting in a similar manner to that of the unit cell). In this case, the largest change in the unit cell was correlated with a decrease in the length of the *a* axis from 82.5 Å in cluster A to 82.1 Å in cluster B (Fig. 2 ▸ *f*). Although the *a* axis length in cluster A is greater than in cluster B, there is still a significant overlap between the two groups, showing that the partition in reciprocal space cannot be established by unit-cell dimension alone. The use of the GUI to generate these plots is demonstrated in Fig. 3 ▸.

**Figure 4:**
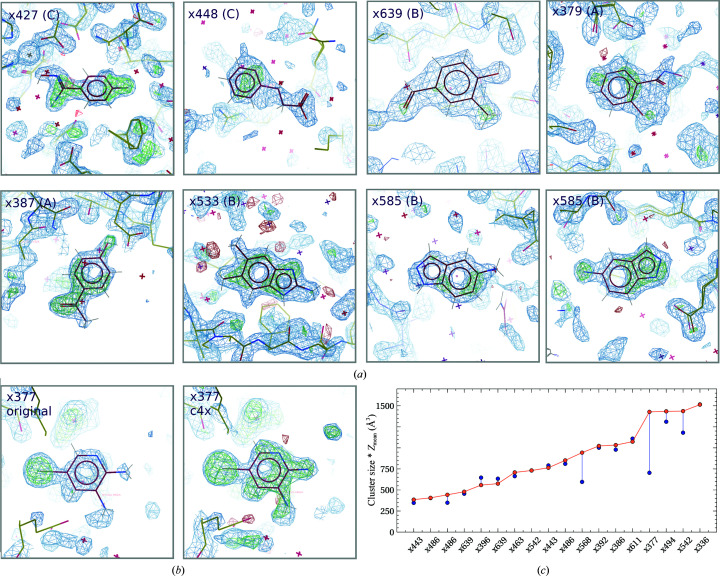
(*a*) Newly identified hits for JMJD2DA showing *PanDDA* maps as displayed in Fig. 2 ▸ labelled with the data-set name and the cluster of which it was a member. (*b*) Left, x377, cluster A from the unpartitioned *PanDDA* analysis, present but not easily interpretable; right, x377, cluster A density using pre-clustering, now easily identifiable as the ligand. (*a*) and (*b*) were rendered in *Coot* (Emsley *et al.*, 2010 ▸). (*c*) Total signal from the *PanDDA* event map, plotted for all data sets shared between the unpartitioned (blue) and pre-clustered (orange) points, ordered by the pre-clustered total signal. All lines are drawn as visual guides to show the change in signal per data set.

JMJD2DA is a larger protein and separated in reciprocal space into three clusters, A (70 data sets), B (43 data sets) and C (108 data sets), associated with small unit-cell shifts (Table 3 ▸) and corresponding real-space changes. Again, separation of the clusters manually was straightforward in reciprocal space and the C^α^ differences were not consulted. However, it is clear from the overlay of all structures that there is no substantial variation in C^α^-atom positions and these variations are small. The enrichment of hits was equally distributed between the clusters. Nevertheless, although they exhibited only small variations of C^α^ positions, running *PanDDA* on the clusters separately did identify nine new hits (three additional hits from cluster A, four from cluster B and two from cluster C; Figs. 4 ▸ *a* and 4 ▸ *b*). One hit from cluster A (x377) was registered in the unpartitioned analysis, but was not sufficiently defined without pre-clustering to be certain of the presence of the ligand (Fig. 4 ▸ *b*).

False negatives can be identified as those which are not shared with the published ligands in the original *PanDDA* study. In JMJD2DA, there were false negatives in both the unpartitioned and pre-clustered analyses: two common to both and four unique to each of the unpartitioned and pre-clustered analyses. The unpartitioned run therefore also missed ligands that had been previously reported. This was owing to the modification of the min_build_datasets parameter. In general, the total signal is either roughly identical or significantly improved by *cluster*4*x* (Fig. 4 ▸ *c*). The average increase of 9.2% is owing to a 16% increase in volume, which is balanced by a reduction of 5.3% in the mean *Z*-value.

**Figure 5:**
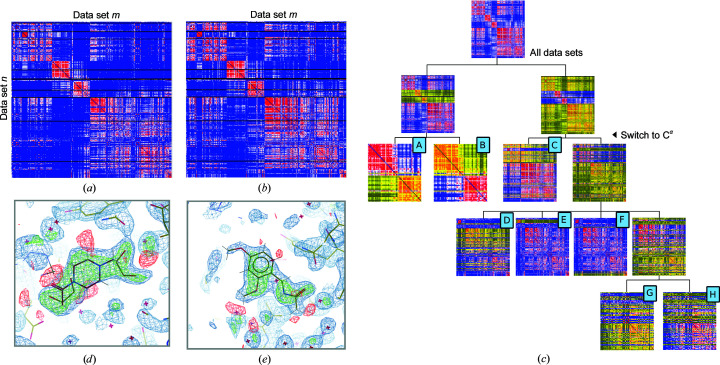
Multi-data set BRD1A. (*a*) Matrix plot showing the relationships between data sets in reciprocal space using the same colour scheme as in Fig. 2 ▸(*a*). (*b*) Matrix plot showing relationships in real space between C^α^ positions. (*c*, *d*) *PanDDA* maps displayed as in Fig. 2 ▸. (*c*) Tree showing the generation of subclusters from all data sets. Selections for subclusters were chosen through inspection of the SVD plots. A subselection of data sets contributes to each matrix plot, and of these a subset is highlighted in yellow, either denoting the final members of clusters A–H or, if a non-terminal cluster, the subselection displayed in the downstream matrix plots. Clusters A and B were split along reciprocal amplitude differences, and clusters C–H were further split along C^α^ differences. (*d*) Newly identified hit from x165. (*e*) Newly identified hit from x324. (*d*) and (*e*) were rendered in *Coot* (Emsley *et al.*, 2010 ▸).

BRD1A is a four-helix bundle protein and the only one of the fragment-screen multi-data sets which showed a clear ordered separation of crystal morphologies according to crystal number, presumably collected chronologically (Fig. 5 ▸ *a*). There is also a strong correlation, as expected, between reciprocal-space variation and real-space variation (Fig. 5 ▸ *b*). The separation was less clear-cut in reciprocal space alone, and so a broad separation into three larger groups was carried out using amplitude differences followed by C^α^ differences to produce a finer slicing of clusters. The tree showing subclustering outcomes is shown in Fig. 5 ▸(*c*). These separated into eight distinct clusters from 302 data sets, summarized in Table 4 ▸, of which four fell below the default parameter for the minimum number of data sets required to trigger statistical characterization in *PanDDA* (40) and two fell below the number chosen in this analysis (20). Only one group exceeded the threshold recommended for statistical characterization (60).

One of the clusters of 11 data sets yielded considerably higher *R* factors (*R*_work_ = 27.3%, *R*_free_ = 31.4%) compared with the average (*R*_work_ = 20.1%, *R*_free_ = 23.7%) and exhibited a considerable rotation of the protein, along with the largest expansion of the *a* axis by 0.6 Å over the average. Although this brought the average *a* axis within 0.1% of that for the *b* axis and therefore ran the risk of mis-indexing during data reduction, no mis-indexing was detected in reflection ampltidues from individual data sets. Exclusion of these 11 data sets identified from *cluster*4*x* increased the average total signal, as calculated above, by 1.4% and produced one extra event to analyse after running *PanDDA* (87 instead of 86 potential hits). No hits were originally found in these 11 data sets. The second small cluster, a set of ten sequential data sets which appeared to vary similarly to one another and distinctly differently to the rest of the data sets, had no elevation in *R* factor (*R*_work_ = 19.0%, *R*_free_ = 22.6%) but also did not harbour any hits in the original analysis or in a forced *PanDDA* analysis. Overall, for BRD1A the small number of data sets collected and the wide variability in the protein meant that most of the clusters dropped below the threshold for statistical characterization. However, one clean additional hit was detected in a cluster of 25 data sets (Fig. 5 ▸ *c*) and another in the largest cluster of 63 data sets (Fig. 5 ▸ *d*). No hits found in the unpartitioned analysis were missing from the pre-clustered analysis.

## 4. Conclusions   

In this paper, *cluster*4*x* has been applied to drug screens; however, it could be applied to other types of experiment as a separate, unbiased method to validate the presence of a concerted change in signal in the amplitudes as a function of another dimension, such as in time-resolved experiments or those involving static laser-induced or temperature-induced changes.

In all four test cases, pre-clustering was instrumental in identifying new hits and clarifying previous hits, but this was most marked in the highly heterogenous multi-data set PTP1B, which also benefited from a larger number of starting structures, which allowed greater subdivision into clusters. Of the three smaller and more homogenous multi-data sets, the reduction in the number of data sets entering the statistical characterization is a drawback. However, analysing more homogenous clusters of data sets is also a way to enhance the signal to noise in the statistical characterization, and this remains a balancing act. As a result, for more homogenous multi-data sets with clusters which often drop below 60 members, the recommendation would be to run both an unpartitioned and a pre-clustered analysis to capture all fringe hits. Nevertheless, treating all these multi-data sets with pre-clustering did reveal additional hits which otherwise fell below the *Z*-map threshold. Analysis of most multi-data sets would therefore benefit from pre-clustering, if only to be certain that all possible putative hits are being found, despite any residual heterogeneity.

Pressure is now mounting to identify ligands disrupting the function of SARS-CoV-2 (Riva *et al.*, 2020 ▸). Although coronaviruses have large genomes by the standard of RNA viruses, we are limited to a targeting a small number of structural, nonstructural and putative open reading frame proteins in the coronavirus genome with small-molecule inhibitors. The widespread economic and social devastation caused by the SARS-CoV-2 pandemic necessitates an understanding of these protein structures for inhibitor design and discovery as quickly as possible. When a virus is of such global significance, lower quality crystals may still provide an acceptable basis to perform a drug screen in a timely fashion. *cluster*4*x* has already been instrumental in identifying an existing drug, 2-methyl-1-tetralone, which covalently binds to the active site of the main protease (Günther *et al.*, 2020 ▸) and other compounds which have passed at least phase I trials (Günther *et al.*, unpublished work). These successes show how crucial it is to minimize losses of potential hits owing to heterogeneity in crystal systems used in X-ray crystallography drug or fragment screens, and *cluster*4*x* is well placed to address many of the problems caused by crystal-to-crystal fluctuations.

One may argue that some of the main benefits of *cluster*4*x* are the drill-down interactive methods provided by the graphical user interface and the opportunity for researchers to explore and understand the peculiarities of their crystals. *cluster*4*x* is provided as a submodule within the *Vagabond* software suite (https://vagabond.hginn.co.uk). It is written in C++ and published under the GPLv3 software licence.

## Figures and Tables

**Table 1:** Average *R*_work_, *R*_free_, unit-cell dimensions and hit information for the 20 clusters determined by *cluster*4*x* for PTP1B

Cluster	No. of data sets	*R*_work_/*R*_free_ (%)	*a* (Å)	*c* (Å)	Average resolution (Å)	No. of hits	+*cluster*4*x* hits
1	51	18.8/21.7	89.87	106.57	1.79	47	+2
2	98	25.3/27.4	89.81	106.57	1.84	0	+5
3	114	25.6/27.5	89.95	106.63	1.83	0	+5
4	64	24.3/26.9	89.37	106.00	2.02	0	+0
5	104	25.3/27.6	89.67	106.34	1.89	0	+8
6	92	25.5/27.5	90.22	106.89	1.96	0	+1
7	86	25.6/27.6	90.10	106.79	1.89	0	+3
8	45	25.9/28.9	90.86	107.41	2.28	0	+1
9	114	25.7/28.1	90.48	107.01	2.02	0	+14
10	66	18.7/21.6	89.92	106.61	1.79	63	+0
11	73	25.8/28.1	89.83	106.51	1.89	0	+8
12	69	25.8/27.7	89.91	106.61	1.79	0	+5
13	50	26.2/28.9	89.92	106.56	2.06	0	+0
14	57	26.0/28.0	89.72	106.45	1.76	0	+1
15	65	24.6/27.3	89.31	105.98	2.04	0	+1
16	72	24.9/27.3	89.60	106.28	1.95	0	+5
17	114	25.6/27.5	90.11	106.79	1.86	0	+4
18	79	26.0/28.4	90.26	106.83	1.94	0	+2
19	58	25.4/28.7	90.69	107.48	2.43	0	+1
20	89	27.1/29.7	90.55	107.03	2.12	0	+9

**Table 2:** Average *R*_work_, *R*_free_ and unit-cell dimensions for the two clusters determined by *cluster*4*x* for BAZ2BA

Cluster	No. of data sets	*R*_work_/*R*_free_ (%)	*a* (Å)	*b* (Å)	*c* (Å)	Average resolution (Å)	No. of hits	+*cluster*4*x* hits
A	97	18.8/22.2	82.09	96.77	57.96	1.81	7	+0
B	102	18.6/21.7	82.46	96.68	57.98	1.78	2	+2

**Table 3:** Average *R*_work_, *R*_free_ and unit-cell dimensions for the two clusters determined by* cluster*4*x* for JMJD2DA

Cluster	No. of data sets	*R*_work_/*R*_free_ (%)	*a* (Å)	*c* (Å)	Average resolution (Å)	No. of hits	+*cluster*4*x* hits
A	70	15.7/18.8	71.29	150.27	1.55	8	+3
B	43	15.8/18.2	71.69	150.87	1.41	4	+4
C	108	15.6/18.1	71.40	150.32	1.39	18	+2

**Table 4:** Average *R*_work_, *R*_free_ and unit-cell dimensions for the eight clusters determined by *cluster*4*x* for BRD1A

Cluster	No. of data sets	*R*_work_/*R*_free_ (%)	*a* (Å)	*b* (Å)	*c* (Å)	Average resolution (Å)	No. of hits	+*cluster*4*x* hits
A	25	19.9/23.0	55.74	56.61	101.97	1.76	2	+1
B	31	18.5/22.7	55.31	56.37	101.93	2.02	0	+0
C	59	17.9/21.2	55.18	56.26	101.71	1.60	15	+0
D	63	18.5/21.9	55.40	56.49	101.82	1.59	9	+1
E	10	19.0/22.6	55.57	56.17	101.73	1.73	0	+0
F	11	27.3/31.4	56.42	56.38	101.56	2.40	0	+0
G	55	20.6/24.4	55.70	56.51	101.71	1.81	27	+0
H	37	19.1/22.9	55.32	56.46	101.66	1.71	12	+0
